# The Effect of the Ultra-High-Pressure Homogenization of Protein Encapsulants on the Survivability of Probiotic Cultures after Spray Drying

**DOI:** 10.3390/foods8120689

**Published:** 2019-12-17

**Authors:** Kevin E. Mis-Solval, Nan Jiang, Meilin Yuan, Kay H. Joo, George A. Cavender

**Affiliations:** 1 Department of Food Science and Technology, University of Georgia, Griffin, GA 30223, USA; nanjiang6@uga.edu; 2 School of Life Sciences, Jiangxi Science and Technology Normal University, Nanchang 330013, China; yuanmimi@163.com; 3 Department of Food Science and Technology, University of Georgia, Athens, GA 30602, USA; khjoo16@uga.edu

**Keywords:** probiotic encapsulation, high-pressure homogenization, spray drying

## Abstract

Interest in probiotic foods and ingredients is increasing as consumers become more aware of their potential health benefits. The production of these products often involves the use of dry culture powders, and the techniques used to produce such powders often suffer from significant losses of viable cells during drying or require the use of expensive drying technologies with limited throughput (e.g., freeze drying). In this study, the authors examined whether culture survivability during spray drying could be increased via the treatment of two common protein encapsulants with ultra-high-pressure homogenization (UHPH). *Lactobacillus plantarum* NRRL B-1927 (also known as ATCC 10241), a probiotic strain, was suspended in either soy protein isolate (SPI) or whey protein isolate (WPI) which had been either treated with UHPH at 150 Mpa or left untreated as a control. The suspensions were then dried using either concurrent-flow spray drying (CCSD), mixed-flow spray drying (MFSD) or freeze drying (FD) and evaluated for cell survivability, particle size, moisture content and water activity. In all cases, UHPH resulted in equal or greater survivability among spray dried cultures, showed reductions in particle size measures and, except for one marginal case (CCFD SPI), significantly reduced the moisture content of the dried powders. The combination of these findings strongly suggests that UHPH could allow probiotic powder manufacturers to replace freeze drying with spray drying while maintaining or increasing product quality.

## 1. Introduction

As consumers become more aware of the contribution of gut microbiota to human health, the market for probiotic foods and supplements is growing significantly [1]. While people can, and often do, add probiotic cultures to their diet using naturally fermented foods, many rely on formulated products (including supplements) due to issues of taste/preference or a desire for a specific probiotic culture [2,3]. To supply the cultures needed for these products, commercial operations must be able to grow sufficient numbers of bacteria, separate them from the growth medium, and preserve them in some fashion for future use [3]. While freeze drying is used for such preservation, owing to its exceptional ability to preserve cell viability, several factors make it less than ideal, including the length of time needed for drying, the discontinuous nature of the process and the high cost of the process. Though these shortcomings are not as critical when producing cultures for the inoculation of foods for fermentation, if the goal is to fortify a food with high levels of viable probiotic bacteria, or create a nutritional supplement, they can be significant [4]. Another method of preserving probiotic cultures that has been investigated is spray drying. This technology exposes a fine mist of liquid to high velocity hot, dry air in order to quickly drive off water, creating a fine powder that can be readily separated from the airstream via centrifugal separation or other techniques [5]. While rapid, continuous, and inexpensive, the process can greatly reduce probiotic culture counts due to the high temperatures involved [4,6]. One way to limit this problem is to encapsulate the cultures using various biopolymers, which create a protective matrix surrounding cells, greatly increasing survivability. However, even with this technique, significant viable cell losses can be seen [7]. Though many different substances, including polysaccharides, fats, waxes, and various other food ingredients can be used to encapsulate probiotic cultures, dairy derived ingredients are fairly common, providing protection not only during drying, but also during digestion of the encapsulated powders [7,8]. Conventionally, liquid foods can be spray dried under concurrent (CC) and mixed-flow (MX) conditions. According to Cal and Sollohub [9], most heat-sensitive products are spray dried under CC, while MX spray drying allows the production of agglomerated particles in a relative small chamber. Although several authors have reported the microencapsulation of probiotics using CC spray drying [10,11], the effect of MX spray drying on the quality of probiotic powders has not been investigated in detail. 

While dairy products/ingredients provide benefits in encapsulating probiotic cultures, they can pose concerns for product manufacturers. These issues include the potential introduction of milk allergens to non-dairy products, the creation of products unsuitable for those who do not consume dairy products for religious or ethical reasons (e.g., vegans) and, depending on the dairy ingredient used, issues with lactose intolerance. Soy-based ingredients have seen some limited investigation as potential replacements, with Hadzieva, Mladenovska, Crcarevska, Dodov, Dimchevska, Geškovski, Grozdanov, Popovski, Petruševski, Chachorovska, Ivanovska, Petruševska-Tozi, Ugarkovic and Goracinova [6] combining soy protein isolate (SPI) with alginate and discovering that some combinations having excellent survivability, while other saw rates below 30%, while Chávez and Ledeboer [12] found that SPI’s ability to protect cultures during drying varied depending on the drying technique and presence of other compounds, with SPI alone resulting in as low as 28% survival. 

Structural differences in proteins likely explain the performance gap between dairy products and soy products during encapsulation, as protein–protein interactions and protein–cell wall interactions are responsible for the immobilization of bacterial cells [13].

While there may be multiple ways to solve the shortcomings of soy-based encapsulants, the simplest might be to modify their structural components in a manner that would encourage the formation of intermolecular bonds, allowing them to better encapsulate bacterial cells. One technique that has been shown to accomplish this type of modification in other protein-rich ingredients is ultra-high-pressure homogenization (UHPH). This process pumps fluids at pressures in excess of 100 MPa, forcing them through a valve or other pressure release component, exposing them to extreme shear that can reduce particle size [14,15], alter proteins [16], increase viscosity [17] and encourage the formation of intermolecular complexes [18].

This study aimed to examine the effect that subjecting two protein-based encapsulants (SPI and whey protein isolate (WPI)) to UHPH would have on the survivability of probiotic cultures during drying techniques commonly used to produce probiotic powders and to determine whether the magnitude of the effect on that process would differ between the two. Additionally, the authors aimed to characterize any potential difference in the particle size or the moisture content of the dried powders, which should provide some initial insight into future storage issues. It was hypothesized that UHPH treatment would increase the ability of the encapsulants to form various bonds, thereby increasing their protective effect. 

## 2. Materials and Methods 

### 2.1. Materials

Dehydrated peptone, de Man, Rogosa & Sharpe (MRS) broth and MRS agar (Thermo Fisher Scientific, Waltham, Massachusetts, USA) were prepared according to label instructions prior to sterilization. Powdered SPI (ProFam 646, Archer Daniels Midland Company, Chicago, IL, USA) and Powdered WPI (ISOPURE, The Isopure company, Downers Grove, IL, USA) were dissolved in reverse osmosis water to yield 9% suspensions that were stored at 4 °C for 24 h prior to any UHPH or culture suspension in order to ensure full hydration of the powders and provide a sufficiently low inlet temperature during processing. A pure culture sample of *Lactobacillus plantarum*, (NRRL B-1927 aka ATCC 10241) originally isolated from sauerkraut and frequently identified as a probiotic [19,20,21], was obtained from the USDA-ARS Culture collection (Peoria, IL, USA) and used to propagate all cultures used in this study.

### 2.2. Preparation of Probiotic Cultures

Probiotic cultures were propagated by following the conditions recommended by the USDA-ARS Culture collection [22]. First, a loop of culture was transferred into a 9 mL culture tube containing MRS broth. Tubes were capped and incubated at 37 °C for 48 h, before being used to inoculate flasks containing 3 L of MRS broth, which were then incubated for an additional 48 h. Cultures were then stored at 4 °C for up to 24 h prior to centrifugation. After agitation, cultures from a given flask were transferred into 500 mL centrifuge bottles and a refrigerated ultracentrifuge (Sorvall RC-6 plus, Thermo Fisher Scientific, Waltham, MA, USA) fitted with an appropriate rotor (Fiberlite F10-6x500, Thermo Fisher Scientific, Waltham, MA, USA) and kept at 4 °C was used to centrifuge the cultures at 5000× *g* for 15 min. After decanting the supernatant from each bottle, each pellet was re-suspended using 50 mL of 0.1% peptone water. Suspended pellets were then combined and centrifuged again for 10 min to yield a single pellet for encapsulant fortification. 

### 2.3. Preparation of Probiotic Suspensions 

Two suspensions were prepared by mixing 90 g of either SPI or WPI in 1 L of distilled water. Then, the suspensions were UHPH processed at 150 MPa using a dual-intensifier continuous high-pressure homogenizing system (Stansted nm-gen 7900, Stansted Fluid Power, Stansted, England) that had been fitted with a stainless steel metering valve (Model 60vrmm4882, Autoclave Engineers, Fluid Components, Erie, PA, USA) at the outlet and modified to feed from a 6 L vessel that was pressurized with compressed air at approximately 550 kPa. The flow rate was adjusted via the metering valve to 1 ± 0.25 L/min, and the collected samples were refrigerated immediately after collection and allowed to cool to at least 20 °C before any cultures were added. These particular UHPH processing parameters were chosen based on structural/functional effects seen in previous works [17,18,23,24,25] as well as the fact that they fall within the working range of many commonly available UHPH equipment. Encapsulants for control trials were used directly from refrigerated storage. Probiotic pellets were dispersed in control- and UHPH-treated suspensions to produce probiotic suspensions (~10^9^ CFU/mL) which were stored under refrigeration for up to 48 h prior to drying to ensure complete chilling and to facilitate transit between processing locations. 

### 2.4. Drying of Samples

**Figure 1:**
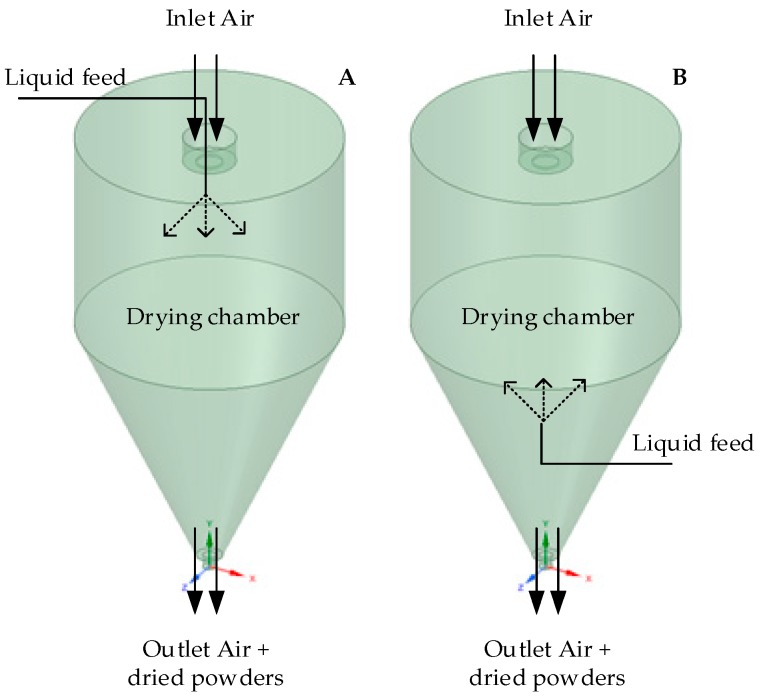
Spray drying configurations: (**A**) concurrent (CC) and (**B**) mixed-flow (MX).

Aliquots (1L) of each probiotic suspension were spray dried (SD) under concurrent (CC) and/or mixed-flow (MX) conditions using a pilot-scale spray drier (Anhydro, PSD 52, Copenhagen, Denmark) at the pilot plant of the University of Georgia (UGA) Food Product Innovation and Commercialization Center (Food PIC-Griffin, GA, USA). In CC spray drying, both drying air and atomized droplets of liquid feed are introduced from the top and exit at the bottom of the drying chamber (DC) of the spray dryer; however, in MX spray drying, drying air enters the DC from the top and the liquid feed is atomized from the bottom. Both exit at the bottom of the DC (Figure 1). Most heat-sensitive products are dried under CC configurations, while heat-stable products are processed in MX drying designs [9]. In both CC and MX, the inlet temperature was set at 140 °C and outlet was kept at 80 ± 2 °C. The feed flow rate was set between 1.5 and 1.75 L/h. Drying conditions were selected based on work previously performed on *Lactobacillus plantarum* strains using various encapsulants [10,26,27]. Concurrently, the probiotic suspensions were also freeze dried (FD). Freeze drying was carried out by freezing 1 L of probiotic suspensions for 12 h. Frozen samples were loaded into pilot-scale lyophilizer (Virtis, the Virtis Company, Gardiner, NY, USA) and dried at −55 °C for 72 h to produce freeze-dried powders. Freeze-dried samples were milled using a high-performance blender (Vitaminix 7500, Olmsted Township, Olmsted Township, OH, USA). Dried powders were stored at −20 °C in a desiccator until the day of analyses (no more than 48 h for enumeration and no more than 5 days for physical properties), and all trials were performed in triplicate.

### 2.5. Enumeration of Probiotic Cultures

Cell counts of *L. plantarum* (NRRL B-1927) were quantified in probiotic suspensions and dried powders by following a modified method described by Reyes, Chotiko, Chouljenko, Campbell, Liu, Theegala and Sathivel [10]. Briefly, 0.1 mL of probiotic suspensions or 1 g of dried powders were serially diluted in sterile Butterfield’s phosphate buffer and 0.1 mL of each dilution was pour plated using 100 mm diameter plates and MRS agar. Dried powders were allowed to fully hydrate in the buffer solution prior to dilution to ensure homogeneous samples. All plating was performed in triplicate, and plates were incubated for 48 h at 37 °C under aerobic conditions before cell colonies were counted. For ease of comparison, results were expressed as the Log of colony forming units (CFU) per gram of dried solids (Log CFU/g).

### 2.6. Physical Properties of Dried Powders

#### 2.6.1. Moisture Content and Water Activity (a_w_)

The moisture content of the powders was determined by moisture analyzer (HR73 Halogen Moisture Analyzer, Mettler-Toledo GmbH, Greifensee, Switzerland). Water activity (a_w_) values were obtained by using a water activity meter (AquaLabSeries 4 TE, Decagon Devices, Inc., Pullman, WA, USA). 

#### 2.6.2. Particle Size Distribution

The particle size distribution of powdered samples was tested using an automated particle size analyzer equipped with laser diffraction (Model PSA 1190, Anton Paar GmbH, Graz, Austria). Briefly, dried powders were placed into the unit’s feed hopper and transported via Venturi/free fall to the analytical area where they were automatically illuminated with three lasers from low to high angles and the diffracted light was analyzed by the system. The whole light scatter pattern was collected and used to calculate the particle size distribution using the Modified Michelson Interferometer (MIE) method which quantifies the angular distribution of backscattered light. The results were reported for D10, D50, and D90 which are the volume diameters of the particles at 10%, 50%, and 90% cumulative volume respectively and the span value (spread of particles) was calculated by following the method described by Mis Solval, et al. [28].

### 2.7. Statistical Analysis

The statistical significance of the observed differences among the means of experimental results was determined by Analysis of Variance (ANOVA) using RStudio statistical software version 1.1.463 (RStudio, Inc. Boston, MA, USA) and followed by post-hoc Tukey’s studentized range tests at an alpha of 0.05. Pearson’s bivariate correlation was used to evaluate the correlations between cell survival in spray and/or freeze-dried powders and particle size distribution values. 

## 3. Results

### 3.1. Probiotic Survivablity 

Probiotic counts before and after drying are presented in Table 1. In all cases, the UHPH-treated encapsulants were either better or equally good at protecting the probiotic cultures during drying than the NO-UHPH-treated encapsulants, and among the spray dried samples, survivability exceeded the rates seen during freeze drying with untreated encapsulants. 

In the case of the NO-UHPH-treated samples, higher cell survival was obtained in MX powders compared to CC and freeze dried (FD) powders and with WPI than with SPI powders. For the UHPH-treated encapsulants, significantly (*p* < 0.05) higher cell survival was also observed in the WPI than in the SPI samples. However, same cell survival was observed in WPI dried in CC, MX and/or FD. Interestingly, major improvements in cell survival were observed in the UHPH-treated encapsulants dried in CC SD and FD. 

### 3.2. Particle Size Distribution

Particle size distribution data are presented in Table 2. It was seen that NO-UHPH-treated WPI Spray Dried samples had smaller particle sizes compared to NO-UHPH-treated SPI SD powders. However, the same effect was not seen in UHPH-treated samples. Of note, UHPH was found to decrease particle size distribution (D_10_, D_50_, and D_90_) for every drying condition and encapsulant, except for freeze-dried SPI. UHPH also slightly increased the spread of the particle size distribution for all drying conditions and encapsulants, except for WPI in MXSD. Furthermore, in the spray dried samples, UHPH caused drastic reduction in both D10 and D90 measures.

#### Effect of Particle Size and Survival of Probiotic Cells

Due to the differences in the production of spray dried and freeze-dried probiotic powders, the correlation analysis between the particle size distribution values of SD powders and cell survival was conducted independently from that of FD powders. 

a. Spray Dried Powders

**Figure 2:**
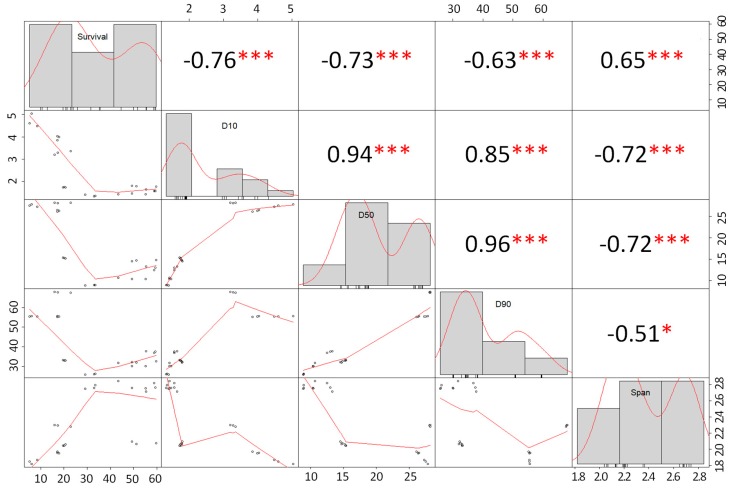
Analysis of correlation between particle size distribution (D10, D50, D90, and span) vs. the survival of probiotic cells in spray dried powders (units, survival = %, D10, D50, D90 = µm). The distribution of each variable is presented in the diagonal. On the left of the diagonal, the bivariate scatter plots with a fitted line are shown. The output numbers represent the correlation coefficient. The stars represent the *p*-value of the correlations: * *p* < 0.05, ** *p* < 0.01, and *** *p* < 0.001.

The analysis of correlation, shown in Figure 2, indicated that there was a highly significant (*p* < 0.05) negative correlation between cell survival and particle size distribution values (D_10_, D_50_, D_90_, and span) which suggests that the smaller the particle size of the spray dried powders, the higher the survival of the probiotic cells (up to certain point). Moreover, there was a significant (*p* < 0.05) positive correlation (0.65) between the span value and the cell survival of probiotic cells. Higher span values are observed in agglomerated particles (powders produced under MX conditions). Hence the results suggest that the higher the span value, the higher the cell survival. 

b. Freeze-Dried Powders

**Figure 3:**
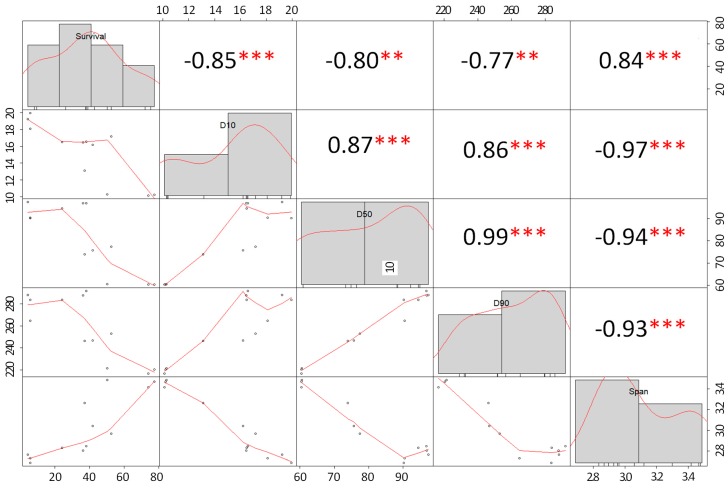
Analysis of correlation between particle size distribution (D10, D50, D90, and span) vs. the survival of probiotic cells in freeze-dried powders (units, survival = %, D10, D50, D90 = µm). The distribution of each variable is presented in the diagonal. On the left of the diagonal, the bivariate scatter plots with a fitted line are shown. The output numbers represent the correlation coefficient. The stars represent the *p*-value of the correlations: * *p* < 0.05, ** *p* < 0.01, and *** *p* < 0.001.

According to the analysis of correlation between cell survival (%) and particle size distribution values (D_10_, D_50_, D_90_, and span) of freeze-dried powders, there was also a highly significant (*p* < 0.05) negative correlation between the variables (Figure 3). These results may suggest that the smaller the particles, the higher the cell survival (%). Furthermore, there was a significant (*p* < 0.05) positive correlation between cell survival and span. Similar results were observed for spray dried powders. It is important to notice that the particle size of the freeze-dried powders is affected by the grinding time/conditions [29]. Hence, the milling conditions for freeze-dried samples were previously standardized in a preliminary study, and 50 g of the grams/batch of material was milled for 1 minute at room temperature.

### 3.3. Moisture Content and Water Activity

Moisture content and particle size data are presented in Table 3. For every drying technology and encapsulant, UHPH reduced the moisture content of the powders significantly, and in several cases, the moisture content saw reductions approaching or exceeding 50%. Reductions in water activity were overall much more modest. 

## 4. Discussion

### 4.1. Survivability of L. plantarum NRRL B-1927 Powders

The increase in survivability seen between the untreated and UHPH-treated encapsulants supports the hypothesis that UHPH alters the encapsulant, allowing it to adopt a more protective configuration, thereby better protecting the probiotic cells. While the exact nature of the structural changes are beyond the scope of this work, previous studies do offer some insight on the potential phenomenon involved. Cavender and Kerr [17] suggested that an interaction between proteins and carbohydrate stabilizers was responsible for profound changes in the viscosity of the ice cream mix and Laneuville, et al. [30] created whey protein/polysaccharide complexes with increased viscosity using UHPH and attributed some of the effect on the physical disruption of tightly bound fibrous complexes. Zamora, Ferragut, Jaramillo, Guamis and Trujillo [18] examined the effect of UHPH on the cheese-making properties of milk, finding that at lower pressures (<230 MPa), curds formed more readily and those curds tended to hold more moisture, strongly suggesting conformational changes in the constituents were allowing for more and/or better interactions. Similar effects have also been seen in soy yogurt that was made from UHPH-treated soymilk [24], and SPI-based emulsions [25], with the latter study suggesting the homogenization increased protein–protein interactions via the hydrophobic effect. Looking at a different legume proteins, Dong, Zhao, Yang, Yang, Shi and Jiang [23] noted increases in water-holding capacity, emulsifying activity and foaming capacity in a peanut protein isolate that had been treated with high-pressure homogenization (pressures ≤80 MPa).

Previous work examining the interaction of encapsulants with the entrained bacterial cells also offer some potential insight into the potential changes that occur to the encapsulants during UHPH. For example, Gong, Di, Yi, Sun, Zhang and Han [13] examined the protective effects of SPI and milk basic proteins (the basic protein fraction of WPI) with and without treatment with transglutaminase, and suggested that electrostatic interaction, particularly between positively charged protein sidechains and bacterial cells, resulted in increased survival. If a similar effect is responsible for the increased survival seen in this study, it could be due to conformational changes brought about by UHPH exposing more positively charged areas, or cleavage of the proteins into subunits with differing overall charges—some of which were positive. Soukoulis, et al. [31] suggests that whey proteins provide antioxidant properties due to sulfur-containing amino acids and are able to prevent lethal oxidative membrane damage during drying. This could help explain the lack of improvement in survivability during MXSD and FD with WPI—in these more gentle drying technologies, the primary cause of cell death might be membrane oxidation, and as the antioxidant sulfur-rich amino acids lie primarily near the surface in β-Lactoglobulin [32] (the dominant protein in WPI), cleavage or denaturation due to UHPH would not change the ability of those side chains to interact with oxygen species. This might also help explain the differences in WPI and SPI in MXSD and FD, as soy proteins are well known to be low in sulfur-rich amino acids [33,34,35] and the native conformation of soy proteins relies upon disulfide bridges to stabilize the tertiary structure [36].

In the current study, profound increases in survivability due to UHPH were seen in all of the encapsulant/drying technology combinations, except for MXSD and FD with WPI as the encapsulants—for which, there was no statistically significant change in survival. It is important to note that those combinations had the highest survival rates in the non-UHPH samples, and so they may represent the practical limit of survivability of the strain during drying in general. The explanation behind the observed results is likely due to changes in protein size and/or conformation. This is similar to the findings of Guraya and James [37], who showed that rice proteins became more soluble and starch granules became smaller after UHPH. The difference in survivability change due to UHPH between the two spray drying technologies is likely related to differences in the two techniques. MXSD is thought to be more gentle, as CCSD exposes liquid product to higher initial temperatures, which may injure bacterial cells more profoundly [5]. Thus, the need for a more protective encapsulant is lower in MXSD, and so much of the improvement in SPI functionality is wasted there. In CCSD, however, a 300% increase in survivability was seen, and both the UHPH WPI and UHPH SPI samples had survivability greater than that of freeze drying with untreated isolates, which may be enough to justify the use of UHPH-treated encapsulants. 

Survivability during freeze drying of the untreated samples was interesting, as SPI showed survival rates equal to or lower than the spray drying methods, while untreated WPI showed the highest survivability during freeze drying. Interestingly, treating SPI with UHPH seems to remedy the deficiency seen during freeze drying, as the treated samples showed the greatest increase in survivability—over 600%. While this might seem promising, the combination of the two technologies (freeze drying and UHPH) would likely be cost prohibitive for manufacturers, and the overall survivability of those samples was equal to or less than that of the UHPH spray dried samples. 

### 4.2. Particle Size

Particle size is an important property that can affect the practical functionality of powdered ingredients, influencing dispersibility, mouthfeel and appearance [38,39]. It has also been suggested that larger particles are better able to protect probiotic cells, at the expense of dispersibility [39]. While this may initially suggest that the untreated FD samples would perhaps have the best survival, the survivability data does not bear this out—FD SPI had the same particle size in the control and UHPH samples, but the survivability differences between the two were profound. Instead, it is most likely that the particles seen in the FD samples, both treated and untreated, are likely agglomerations of smaller particles, created due to the bulk nature of freeze drying, while the SD methods, which dry individual droplets would be expected to produce finer particles. When looking at the effect of UHPH on particle size, the fact that particle sizes tended to decrease when treated encapsulants were used is not entirely surprising—not only do multiple studies show the technology can reduce particle sizes [14,15,40,41], but there is also the likelihood that the conformational changes made to the proteins open up additional sites of interaction, thereby allowing the formation of capsules with fewer proteins. Confirming this would require advanced microscopic techniques, which the authors are already pursuing for future publication. 

Moreover, our observations are in agreement with those published by Würth, et al. [42] who reported an inverse (negative) correlation between the mean particle size (D_50_) of spray dried skim milk powders produced at 155 °C and the survival of *Lactobacillus paracasei* ssp. The authors reported survival rates (%) of probiotic cells above 50% in particles with a D_50_ ≈ 5 µm; while powders with a D_50_ ≈ 10 µm had survival rates below 10%. In general, MX powders and UHPH-treated powders had smaller particles sizes than CC- and NO-UHPH-treated powders, respectively. Interestingly, powders with small particle sizes had higher cell survival rates (especially in spray dried powders). This effect may be because bigger particles have longer residence times inside the dryer chamber and, therefore, suffer higher thermal degradation. It is important to note that our analysis of correlation does not prove causation; rather, it is a tool to understand the complexity of the data. Therefore, the high survival rates (%) observed in our study may not be attributed to the particle size of the powders solely.

### 4.3. Moisture Content and Water Activity

The moisture content and water activity of dried probiotic powders are important factors in predicting stability during storage, with higher values correlated with poor survivability [12,43,44,45,46]. In fact, it has been suggested that 5% is the critical moisture content for long-term stability, with values higher than this being correlated with poor stability [12]. In the current study, the spray dried WPI control samples had moisture activities above this value (CCSD) or within one standard deviation of it (MXSD), while the SPI control samples were slightly below the value. The UHPH samples which were spray dried were all below the critical value, indicating the technology could be used to ensure the production of consistent low-moisture probiotic powders. FD samples showed more profound changes in moisture content, but all were far below the critical value. 

The limited or non-existent decreases in water activity between the control and UHPH samples suggest that the moisture differences seen are likely a result of losses in free water, rather than bound water. Further, while one of the freeze-dried samples did show an increase in mean A_w_ of 0.01 units, both this difference and the measures of A_w_ upon which it was determined are close to the technical limits of the instrument. For all samples, the A_w_ measures were sufficiently low to prevent the growth of undesirable microbes, and would result in nearly non-existent enzymatic activity [47]. Further, while these levels are known to encourage lipid oxidation, the lack of lipids in the encapsulants should also render that concern moot as well. Thus it can be assumed that, when properly packaged and stored, all of the probiotic powders produced in this study should have a reasonably long shelf life, and a study is currently underway to better quantify this and to determine what effect, if any, UHPH treatment has on storage stability. 

## 5. Conclusions

UHPH provides interesting potential to improve the encapsulating ability of protein isolates during spray drying, reducing particle sizes, moisture content and improving survivability. The study also demonstrated that probiotic powders with high cell survival can be effectively produced by mixed-flow spray drying. In particular, for UHPH-treated a soy protein isolate allows the use of concurrent spray drying, the most common type of spray drying, to produce probiotic powders that have no dairy-related concerns with markedly lower culture loss than the more expensive and slower freeze drying process. While the technology clearly can improve survival during the spray drying process, it remains to be seen what effect the process will have on culture survival during storage and, perhaps most importantly, during digestion. While it is likely that the changes in the encapsulants which are responsible for protecting the bacteria during drying will also protect them during storage and digestion, it is possible that the conformational changes might have a deleterious effect, and determining whether this is the case is important before a recommendation to the industry can be made. Therefore, in addition to investigating the effects on other probiotic strains and optimizing drying conditions, future studies should also be aimed at these important questions.

## Figures and Tables

**Table 1:** Cell counts (Log (CFU/g solids)) of L. plantarum (NRRL B-1927) encapsulated in ultra-high-pressure homogenized soy protein isolate (SPI) and/or whey protein isolate (WPI) suspensions after spray drying.

Drying Method	Encap. Mat.	NO-UHPH			UHPH (150 Mpa)		
		Log CFU/g Solids		Cell Survival (%)	Log CFU/g Solids		Cell Survival (%)
		Before Drying	After Drying		Before Drying	After Drying	
CC	SPI	8.67 ± 0.32	8.43 ± 0.08	6.56 ± 1.75 ^d,B^	9.27 ± 010	8.47 ± 0.17	20.10 ± 0.56 ^d,A^
CC	WPI	9.22 ± 0.02	8.46 ± 0.01	17.37 ± 0.37 ^c,B^	9.20 ± 0.08	8.93 ± 0.04	53.39 ± 5.58 ^a,b,A^
MX	SPI	9.16 ± 0.30	8.43 ± 0.08	18.68 ± 3.66 ^c,B^	9.27 ± 010	8.77 ± 0.03	31.86 ± 2.39 ^c,d,A^
MX	WPI	9.02 ± 0.02	8.78 ± 0.02	57.87 ± 2.16 ^a,A^	9.20 ± 0.08	8.87 ± 0.10	49.19 ± 5.90 ^a,b,c,A^
FD	SPI	9.16 ± 0.30	7.87 ± 0.07	5.13 ± 0.81 ^d,B^	9.27 ± 010	8.78 ± 0.11	32.77 ± 7.65 ^b,c,d,A^
FD	WPI	9.02 ± 0.02	8.66 ± 0.08	43.87 ± 7.98 ^b,A^	9.20 ± 0.08	9.02 ± 0.10	67.29 ± 14.97 ^a,A^

Means ± Standard Deviation (SD). *n* = 3. Survival rates followed by different miniscule (a, b, c, etc.) letters in the same column are significantly different (*p* < 0.05). Survival rates followed by different majuscule (A, B, C, etc.) letters in the same row are significantly different (*p* < 0.05). CC = concurrent spray drying, MX = mixed-flow spray drying; FD = freeze drying; SPI = soy protein isolate; WPI = whey protein isolate.

**Table 2:** Particle size distribution values of dried probiotic powders containing *L. plantarum* (NRRL B-1927).

Drying Method	Encap. Mat.	NO-UHPH				UHPH (150 Mpa)			
		Particle Size Distribution				Particle Size Distribution			
		D_10_ (µm)	D_50_ (µm)	D_90_ (µm)	Span	D_10_ (µm)	D_50_ (µm)	D_90_ (µm)	Span
CC	SPI	4.71 ± 0.29 ^c,A^	27.54 ± 0.26 ^c,A^	55.52 ± 0.13 ^c,A^	1.85 ± 0.02 ^d,B^	1.74 ± 0.02 ^c,B^	15.30 ± 0.10 ^c,B^	33.09 ± 0.09 ^c,B^	2.05 ± 0.01 ^c,A^
CC	WPI	3.96 ± 0.10 ^c,d,A^	26.32 ± 0.17 ^c,A^	55.46 ± 0.23 ^c,A^	1.96 ± 0.01 ^d,B^	1.79 ± 0.01 ^c,B^	14.69 ± 0.15 ^c,B^	32.28 ± 0.25 ^c,B^	2.08 ± 0.02 ^c,A^
MX	SPI	3.28 ± 0.08 ^c,d,A^	28.15 ± 0.05 ^c,A^	67.81 ± 0.17 ^c,A^	2.29 ± 0.01 ^c,B^	1.36 ± 0.03 ^d,B^	8.92 ± 0.02 ^d,B^	26.07 ± 0.22 ^d,B^	2.77 ± 0.02 ^b,A^
MX	WPI	1.59 ± 0.04 ^d,A^	12.94 ± 0.41 ^d,A^	37.39 ± 0.50 ^d,A^	2.77 ± 0.05 ^b,A^	1.44 ± 0.03 ^d,B^	10.47 ± 0.17 ^d,B^	30.66 ± 0.97 ^c,d,B^	2.79 ± 0.05 ^b,A^
FD	SPI	19.09 ± 0.93 ^a,A^	92.62 ± 4.07 ^a,A^	278.85 ± 12.46 ^a,A^	2.73 ± 0.04 ^b,B^	16.50 ± 0.06 ^a,B^	96.02 ± 1.37 ^a,A^	287.91 ± 3.98 ^a,A^	2.83 ± 0.02 ^b,A^
FD	WPI	15.49 ± 2.11 ^b,A^	75.66 ± 1.75 ^b,A^	248.69 ± 3.70 ^b,A^	3.09 ± 0.15 ^a,B^	10.20 ± 0.07 ^b,B^	60.50 ± 0.03 ^b,B^	219.43 ± 2.49 ^b,B^	3.46 ± 0.04 ^a,A^

Means ± SD, *n* = 3. Particle sizes followed by different miniscule (a, b, c, etc.) letters in the same column are significantly different (*p* < 0.05). Particle sizes followed by different majuscule (A, B, C, etc.) letters in the same row for a given measure (D_10_, D_50_, span, etc.) are significantly different (*p* < 0.05). CC = concurrent spray drying, MX = mixed-flow spray drying; FD = freeze drying; SPI = soy protein isolate; WPI = whey protein isolate; Dx = xth percentile particle size.

**Table 3:** Moisture content and water activity values of UHPH-treated powders containing *L. plantarum* (NRRL B-1927).

Drying Method	Encapsulating Material	NO-UHPH		UHPH (150 Mpa)	
		Moisture (%)	Water Activity (a_w_)	Moisture (%)	Water Activity (a_w_)
CC	SPI	4.35 ± 0.09 ^b,A^	0.22 ± 0.00 ^a,B^	3.81 ± 0.43 ^a,B^	0.23 ± 0.00 ^a,A^
CC	WPI	5.18 ± 0.16 ^a,A^	0.22 ± 0.00 ^a,A^	3.89 ± 0.42 ^a,B^	0.21 ± 0.01 ^a,B^
MX	SPI	4.16 ± 0.22 ^b,A^	0.20 ± 0.00 ^b,A^	2.25 ± 0.11 ^b,A^	0.15 ± 0.01 ^b,B^
MX	WPI	4.83 ± 0.25 ^a,A^	0.22 ± 0.00 ^a,A^	3.79 ± 0.07 ^a,B^	0.21 ± 0.02 ^a,A^
FD	SPI	1.03 ± 0.05 ^d,A^	0.03 ± 0.00 ^d,A^	0.41 ± 0.07 ^c,B^	0.04 ± 0.00 ^c,A^
FD	WPI	2.04 ± 0.12 ^c,A^	0.06 ± 0.00 ^c,A^	0.24 ± 0.09 ^c,B^	0.03 ± 0.00 ^c,B^

Means ± SD, *n* = 3. Particle sizes followed by different miniscule (a, b, c, etc.) lettera in the same column are significantly different (*p* < 0.05). Particle sizes followed by different majuscule (A, B, C, etc.) letters in the same row for a given measure (moisture or water activity) are significantly different (*p* < 0.05).
